# Cannabis smoking is associated with persistent epigenome-wide disruptions despite smoking cessation

**DOI:** 10.1186/s12890-025-03634-9

**Published:** 2025-04-09

**Authors:** Ana I. Hernandez Cordero, Xuan Li, Chen Xi Yang, Amirtha Ambalavanan, Julie L. MacIsaac, Michael S. Kobor, Dany Doiron, Wan Tan, Jean Bourbeau, Don D. Sin, Qingling Duan, Janice M. Leung, the CanCOLD Collaborative Research Group, Dany Doiron, Jean Bourbeau, Don D. Sin, Wan C. Tan, JMark FitzGerald, Darcy D. Marciniuk, Denis E. O’Donnell, Paul Hernandez, Kenneth R. Chapman, Brandie Walker, Shawn Aaron, François Maltais, Jonathon Samet, Milo Puhan, Qutayba Hamid, James C. Hogg, Palmina Mancino, Pei Zhi Li, Dennis Jensen, Carolyn Baglole, Yvan Fortier, Wan C. Tan, Don Sin, Julia Yang, Jeremy Road, Joe Comeau, Adrian Png, Kyle Johnson, Harvey Coxson, Jonathon Leipsic, Cameron Hague, Miranda Kirby, Mohsen Sadatsafavi, Teresa To, Andrea Gershon, Wan C. Tan, Harvey Coxson, Pei-Zhi Li, Zhi Song, Andrea Benedetti, Dennis Jensen, Yvan Fortier, Miranda Kirby, Wan C. Tan, Christine Lo, Sarah Cheng, Elena Un, Cynthia Fung, Wen Tiang Wang, Liyun Zheng, Faize Faroon, Olga Radivojevic, Sally Chung, Carl Zou, Palmina Mancino, Jacinthe Baril, Laura Labonte, Kenneth Chapman, Patricia McClean, Nadeen Audisho, Brandie Walker, Curtis Dumonceaux, Lisette Machado, Paul Hernandez, Scott Fulton, Kristen Osterling, Denise Wigerius, Shawn Aaron, Kathy Vandemheen, Gay Pratt, Amanda Bergeron, Denis O’Donnell, Matthew McNeil, Kate Whelan, François Maltais, Cynthia Brouillard, Darcy Marciniuk, Ron Clemens, Janet Baran, Candice Leuschen

**Affiliations:** 1 Centre for Heart Lung Innovation, St. Paul’S Hospital and University of British Columbia, Room 166, 1081 Burrard Street, Vancouver, BC V6Z 1Y6 Canada; 2 Edwin S. H. Leong Healthy Aging Program, Faculty of Medicine, University of British Columbia, Vancouver, Canada; 3 Department of Biomedical and Molecular Sciences, School of Medicine, Queen’s University, Kingston, Canada; 4 Centre for Molecular Medicine and Therapeutics, University of British Columbia, Vancouver, Canada; 5 McGill University Health Centre, McGill University, Montreal, Canada; 6 Division of Respiratory Medicine, Department of Medicine, University of British Columbia, Vancouver, Canada; 7 School of Computing, Queen’s University, Kingston, Canada

**Keywords:** Cannabis, Marijuana, Epigenetics, Smoking cessation, Methylation

## Abstract

**Background:**

The use of cannabis has been associated with both therapeutic and harmful effects. As with cigarette smoking, cannabis smoking may affect the epigenetic regulation (e.g., DNA methylation) of gene expression which could result in long term health effects. The study of DNA methylation in cannabis smoking has to date been restricted to young adults and there remains yet no evaluation of whether cannabis smoking cessation can reverse epigenetic disturbances. Here, we aimed to investigate the relationship between genome-wide DNA methylation and cannabis smoking.

**Methods:**

We used peripheral blood from a subset of older adults within the Canadian Cohort of Obstructive Lung Disease (CanCOLD) cohort (*n* = 93) to conduct an epigenome-wide DNA methylation analysis that identified differential methylated positions (DMPs) associated with cannabis smoking at a false discovery rate < 0.05. Using these DMPs, we then identified differentially methylated genes (DMGs) that enriched pathways associated with both former and current cannabis smoking status.

**Results:**

We found DMPs corresponding to 12,115 DMGs and 10,806 DMGs that distinguished the current and former cannabis smoking groups, respectively, from the never cannabis smoking group. 5,915 of these DMGs were shared between the current and former cannabis smoking groups. 50 enriched pathways were also shared between the current and former cannabis smoking groups, which were heavily represented by multiple aging- and cancer-related pathways.

**Conclusions:**

Our findings indicate that in older adults, cannabis smoking is linked with epigenome-wide disruptions, many of which persist despite cannabis smoking cessation. Epigenetic modulation of genes associated with aging and cancer that remains even after quitting cannabis should serve as a caution that there may be long-lasting epigenetic injury with cannabis smoking.

**Trial registration:**

NCT00920348.

**Supplementary Information:**

The online version contains supplementary material available at 10.1186/s12890-025-03634-9.

## Background

Access to cannabis and its derived products has increased due to its legalization in a growing number of countries including Canada and specific regions of the United States [1]. For decades, the use of cannabis, both therapeutic and recreational, has been a controversial topic. While cannabis smoking has been effective at treating nausea and vomiting in patients undergoing chemotherapy [2] and has been proposed as a treatment for chronic pain [3], multiple sclerosis [4], and epilepsy [5], its effects remain inconsistent across studies [6]. On the other hand, cannabis is also associated with an increased risk of psychosis [7] and pregnancy complications [8]. Whether the benefits of cannabis outweigh its health risks remains a subject of ongoing debate.

The methods of cannabis consumption are highly variable across populations as are the proportions of cannabinoids within different varieties, thus the assessment of its impact on health is challenging. As of 2021, smoking was the most common method of cannabis use in Canada, follow by eating and vaporization through e-cigarettes [9]. Cannabis smoking specifically has been associated with increased respiratory symptom burdens [10] and faster lung function decline in older adults [11]. The molecular mechanisms that may increase these risks are not well known, however, we propose in this study that epigenetic dysregulation may shed light on pathological responses to cannabis smoking. DNA methylation is one such epigenetic mechanism, which involves the addition or removal of a methyl group at a cytosine-guanine residue (CpG) site along regions of the genome. These changes are dynamic, responsive to environmental factors and toxins, and can influence downstream gene expression. Although most studies of DNA methylation in cannabis smoking have to date been restricted to young adults [12–14], we have recently demonstrated in a cohort of older individuals that cannabis smoking is associated with accelerated epigenetic aging [15]. However, there remains as yet no evaluation of whether cannabis smoking cessation can reverse epigenome-wide disturbances. Here, we hypothesize that cannabis smoking has a detrimental effect on DNA methylation, even after smoking cessation, and that DNA methylation may represent a mechanistic link between cannabis smoking and adverse health outcomes.

## Methods

### Study cohort

To investigate the effect of cannabis smoking and cannabis smoking cessation on the epigenome we used the Canadian Cohort of Obstructive Lung Disease (CanCOLD) study, a prospective cohort study that recruited males and females aged > 40 years by sampling the population in nine Canadian cities (Vancouver, Saskatoon, Calgary, Toronto, Ottawa, Kingston, Montreal, Quebec City, and Halifax) (ClinicalTrials.gov identifier NCT00920348, Registration Date 2009–06–12) [16]. For this study, we used a subset of participants within the cohort (*n* = 93). The comparisons between the full CanCOLD cohort (*n* = 1,500) [16] and our study subset are shown in Additional file 1. Pre- and post-bronchodilator spirometry were performed according to the American Thoracic Society/European Respiratory Society guidelines [17, 18].

### DNA methylation profiling

Whole blood samples were collected from participants at the baseline study visit using a standard venipuncture protocol. After DNA extraction and bisulfite conversion, these samples were profiled for DNA methylation using the Illumina Infinium MethylationEPIC BeadChip microarray, which interrogates 863,904 DNA methylation sites (CpG probes) across the genome. The samples were profiled at two separate laboratories (subset 1: *n* = 34, subset 2: *n* = 59); raw data were thus processed separately using filtering, quality controls, and normalization steps according to previously described methods that have been standardized by our laboratory [19, 20]. First, we calculated beta values based on the methylation probe intensity for each CpG (ranging from 0 [all unmethylated] to 1 [all methylated]) and transformed these to M-values (log2 ratio of the intensity of the methylated probe to unmethylated CpG probe). Probes were then filtered based on their probe detection quality (p > 1e- 10). XY-linked, non-CpG, single nucleotide polymorphism (SNP), and cross-hybridization probes were also removed. Background correction, normalization, and batch correction were applied to the data using normal–exponential out-of-band [21], mixture quantile normalization [22], and ComBat [23] methods, respectively.

### Epigenome-wide differential methylation analyses

Methylation beta values (the percentage across the sample of each CpG that is methylated) were logit transformed into M values. Beta values were used to calculate cell proportions using the DNA methylation age calculator website (https://dnamage.genetics.ucla.edu/home) based on methods by Houseman et al. [24]. We first calculated ancestry principal components (PC) (PC1 to PC5) in each subset using EPISTRUCTURE software [25] (Additional file 2). We then conducted principal component analysis (PCA) based on DNA methylation by each subset. We used the first two PCs to assess the effect of potential covariates on methylation. To identify DMPs associated with cannabis smoking status, we conducted an epigenome-wide analysis using a robust linear model (rlm) in the MASS R package [26]. We adjusted our model for variables that were either 1) significantly correlated with methylation based on the PCA (for instance, the first two ancestry PCs) or 2) statistically different between the two batches; thus our analysis was controlled for age, sex, cigarette smoking status, cell proportions, and the PCs of ancestry [25]. The full rlm used is shown below:

$$M\;value=Age\;+\;Sex\;+\;Cigarette\;smoking\;status\;+\;Cannabis\;smoking\;status\;+\;PlasmaBlasts\;+\;CD4T\;+\;NK\;cells\;+\;Granulocytes\;+\;PC1\;+\;PC2$$

Since one batch only included females, sex was not included in its analysis. We later combined the subset findings using a meta-analysis implemented in the R package metafor (fixed effects model) [27]. Given the limited sample size of our study cohort, we did not stratify our analyses based on cigarette smoking. We considered significant results based on the following criteria: a significant meta-analysis association at a false discovery rate (FDR) < 0.05 and consistent effects direction (Beta Fold Change [BetaFC]) in both the individual analyses by subset and the meta-analysis. These DMPs were reported and used for downstream analysis.

### Enrichment analyses

We used the R package WebGestaltR [28] over representation analysis to identify Kyoto Encyclopedia of Genes and Genomes (KEGG) pathways that were significantly (FDR < 0.05) enriched by genes that corresponded to DMPs associated with former and current cannabis smoking.

## Results

### Study cohort

Our study cohort consisted of 93 participants from the CanCOLD study and included never (*n* = 51), former (*n* = 32) and current (*n* = 10) cannabis smoking groups; 79% of the former cannabis smoking group reported abstinence over one year before the study. Overall, there were no significant age, body mass index (BMI), lung disease (i.e., chronic obstructive pulmonary disease [COPD] or asthma) or pulmonary function differences between the three groups (all *p* > 0.05) (Table 1). There was a significant difference in the number of individuals who smoked cigarettes (*p* < 0.001), cannabis joint-years (*p* = 0.002), and males (*p* = 0.022) between the groups.

**Table 1:** Study cohort overview

**Variable**	**Cannabis smoking status**			
	**Never (*n* = 51)**	**Former (*n* = 32)**	**Current (*n* = 10)**	***p*-value**
Age (years)	55.15 (48.06–62.60)	57.94 (49.30–60.88)	49.30 (47.41–54.98)	0.255
Female sex, n (%)	42 (82%)	25 (78%)	4 (40%)	0.022
BMI (kg/m^2^)	28.02 (25.45–31.03)	27.05 (23.65–30.55)	27.03 (20.57–28.62)	0.364
Cannabis joint-years	NA	1 (0.06—2.47)	14 (8.01—36.47)	* 0.002
Tobacco cigarette smoking status				< 0.001
**Never,** n (%)	35 (69%)	10 (31%)	1 (10%)	
**Former,** n (%)	3 (6%)	3 (9%)	5 (50%)	
**Current,** n (%)	13 (25%)	19 (59%)	4 (40%)	
COPD, n (%)	11 (26%)	7 (22)	3 (30%)	0.822
Asthma, n (%)	20 (39%)	13 (41%)	4 (40%)	~ 1
Post-bronchodilator FEV_1_% Predicted	87.47 (76.64—95.64)	83.70 (69.23—93.59)	72.24 (65.49—93.06)	0.521
Post-bronchodilator FVC % Predicted	99.12 (88.92—107.09)	98.49 (84.31—108.61)	91.98 (85.66—104.14)	0.663
Post-bronchodilator FEV_1_/FVC (%)	86.20 (79.34—89.98)	85.31 (75.55—90.84)	83.49 (72.76—88.11)	0.735

COPD and asthma were ascertained by self-reported physician diagnoses. Cannabis joint-years = number of joints per day x number of years smoked. Kruskal–Wallis and Fisher tests were used to calculate p-values. *Only the former and current cannabis groups were use in this test

*Abbreviations*: *BMI* body mass index, *COPD* chronic obstructive pulmonary disease, *FEV1* forced expiratory volume in 1 s, *FVC* forced vital capacity. Stats correspond to median and interquartile range (IQR) or percentage (%)

### Cannabis smoking is characterized by significant epigenome-wide alterations

**Fig. 1:**
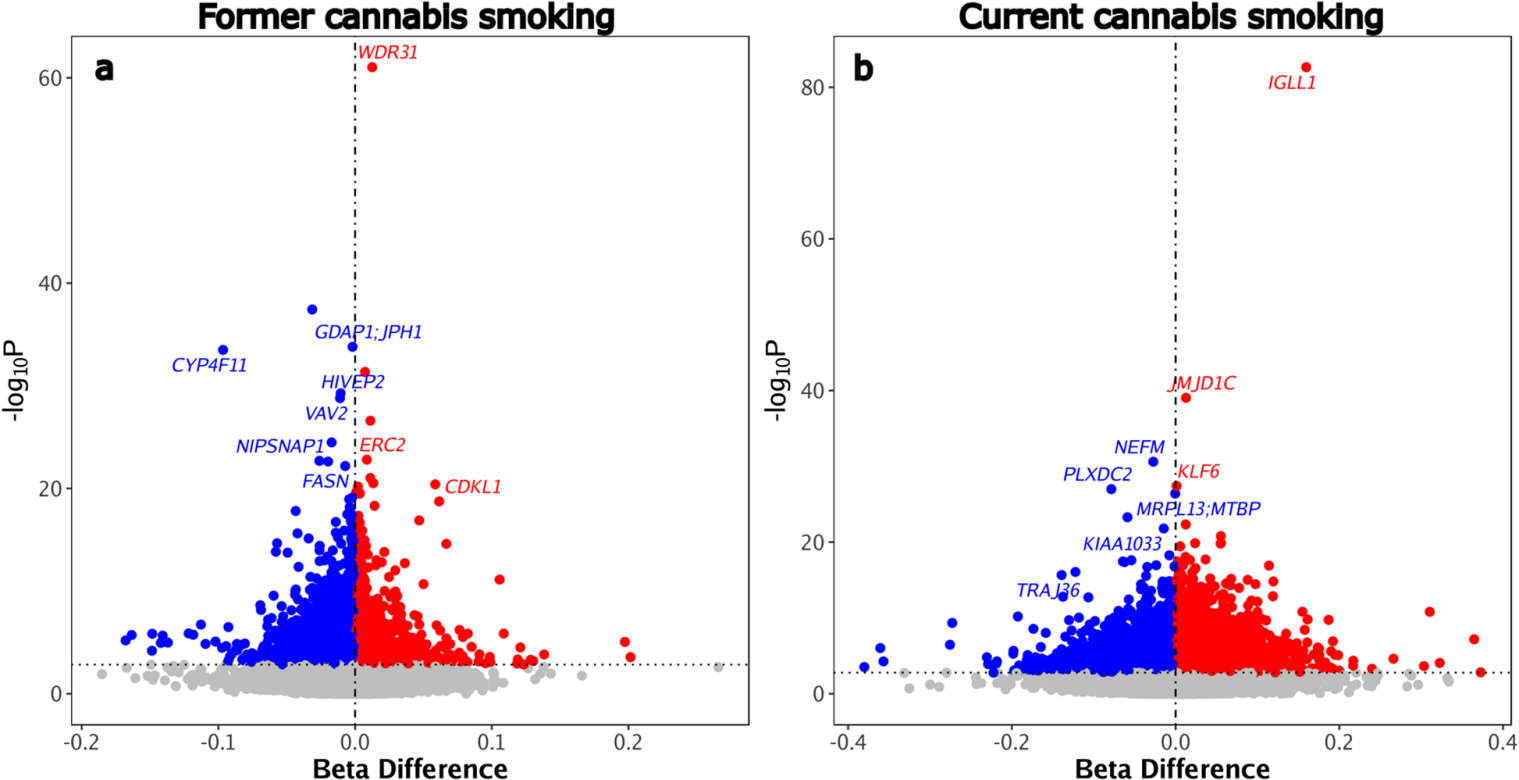
Differentially methylated positions (DMPs) associated with cannabis smoking. Volcano plots are shown for significant DMPs associated with **a** former cannabis smoking and **b** current cannabis smoking with never smoking status as the reference group. Compared to never smoking, hypomethylated DMPs are shown in blue and hypermethylated DMPs are shown in red. The x-axis represents the effect size for each CpG tested, where 0 represents 0% difference in methylation between groups, and 1 represents a 100% methylation difference

We first explored epigenome-wide differential methylation using a meta-analysis approach. Figure 1a shows the 21,176 differentially methylated CpG positions (DMPs) within the vicinity of 12,115 genes (differentially methylated genes [DMGs]) that were associated with former cannabis smoking compared to never smoking, while Fig. 1b shows the 19,819 DMPs (corresponding to 10,806 DMGs) that were associated with current cannabis smoking compared to never smoking. A full list of these DMPs and genes is provided in Additional file 3. Out of the total, 20 (former) and 339 (current) DMPs had effect sizes of ≥ 10% change in methylation compared with the never smoking group. Overall, the effects on the epigenome were larger in the current cannabis smoking (Median BetaFC = 0.011 [0.004–0.025]) compared to former cannabis smoking (Median BetaFC = 0.003 [0.001–0.009]) (Additional file 4). In addition, the distribution of the effects in both in current and former cannabis smoking show over-dispersed distribution (Additional file 4); however lambda values were less than 1, suggesting no significant inflation of the analyses (Additional file 4). Table 2 shows the top DMPs and corresponding genes identified in our analyses, including *WDR31, GDAP, JPH1,* and *CYP4 F11* for former cannabis smoking and *IGLL1, JMJD1 C, NEFM, KLF6,* and *PLXDC2* for current cannabis smoking. Furthermore, 5,915 DMGs were shared between the former and current cannabis smoking groups.

**Table 2:** Top differentially methylated positions associated with former and current cannabis smoking

**CpG**	**Beta difference**	**FDR**	**Chr**	**Relation to CpG Island**	**Gene symbol**	**Cannabis smoking group**
cg14190196	0.012	*7.32* × *10*^*–56*^	9	North Shelf	*WDR31*	Former
cg24960778	− 0.031	*1.45* × *10*^*–32*^	2	Open Sea	No annotation	Former
cg08949296	− 0.002	*4.17* × *10*^*–29*^	8	Island	*GDAP;JPH1*	Former
cg08670281	− 0.097	*6.28* × *10*^*–29*^	19	South Shore	*CYP4 F11*	Former
cg07305270	0.007	*7.03* × *10*^*–27*^	1	Open Sea	No annotation	Former
cg19558972	0.160	*1.79* × *10*^*–77*^	22	Open Sea	*IGLL1*	Current
cg08222002	0.013	*3.65* × *10*^*–34*^	10	South Shore	*JMJD1 C*	Current
cg07502389	− 0.027	*6.52* × *10*^*–26*^	8	Island	*NEFM*	Current
cg26538214	0.001	*7.58* × *10*^*–23*^	10	Island	*KLF6*	Current
cg07413747	− 0.078	*1.61* × *10*^*–22*^	10	Open Sea	*PLXDC2*	Current

*Abbreviations*: *CpG* Cytosine-guanine residue, *FDR* false discovery rate

**Fig. 2:**
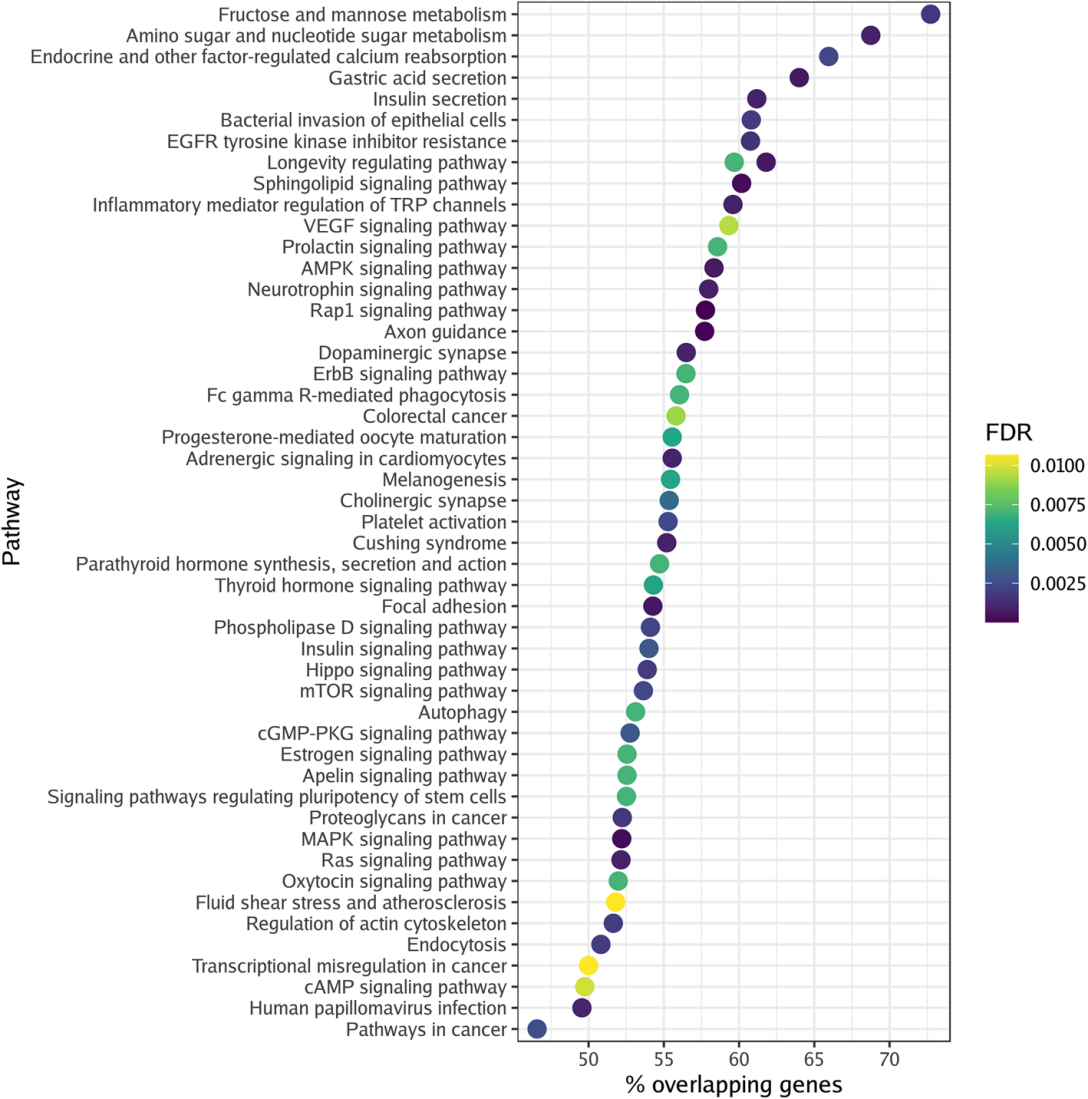
Differentially methylated pathways in former cannabis smoking. The top 50 enriched KEGG pathways are shown for the former cannabis smoking group. Color intensity (black to yellow) represents the level of significance. Abbreviations: false discovery rate – FDR; Kyoto Encyclopedia of Genes and Genomes – KEGG

**Fig. 3:**
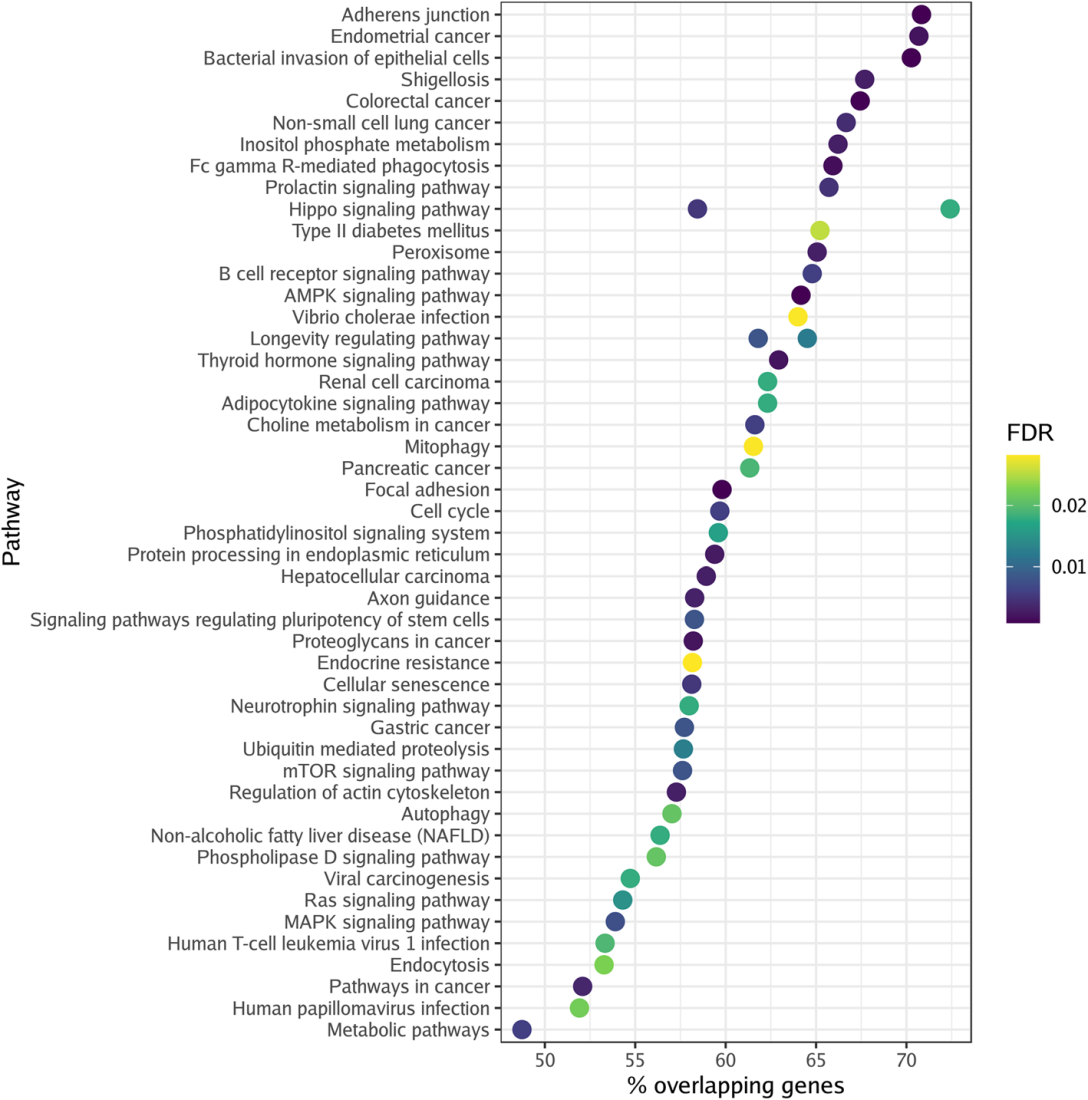
Differentially methylated pathways in current cannabis smoking. The top 50 enriched KEGG pathways are shown for the current cannabis smoking group. Color intensity (black to yellow) represents the level of significance. Abbreviations: false discovery rate – FDR; Kyoto Encyclopedia of Genes and Genomes – KEGG

DMGs enriched 72 pathways (Fig. 2) in the former cannabis smoking group, while 92 pathways were associated with the current cannabis smoking group (Fig. 3). A full list of the pathways can be found in Additional file 5. We identified 50 pathways that overlapped between the former and current cannabis smoking groups (Table 3). These included aging-related pathways such as cellular senescence, insulin resistance, and AMPK, MAPK, mTOR, PI3 K-Akt, and Rap1 signaling. Cancer-related pathways were also heavily enriched in both groups, including choline metabolism in cancer, colorectal cancer, endometrial cancer, gastric cancer, hepatocellular carcinoma, glioma, non-small cell lung cancer, pancreatic cancer, ErbB signaling, Ras signaling, FoxO signaling, pathways in cancer, and proteoglycans in cancer. Unique pathways identified in the former cannabis smoking group included metabolic, peroxisome, and ubiquitin proteolysis pathways. Pathways unique to the current cannabis smoking group included cortisol, dopamine, and oxytocin pathways.

**Table 3:** Overlapping differentially methylated pathways. KEGG pathways characterized by differential methylated genes in both former and current cannabis smoking compared to never smoking

**Pathway**	**FDR current**	**FDR former**
Rap1 signaling pathway	1.247 × 10^–05^	0.034
Axon guidance	7.123 × 10^–05^	0.003
MAPK signaling pathway	2.365 × 10^–04^	0.008
Sphingolipid signaling pathway	2.365 × 10^–04^	0.039
Focal adhesion	0.001	0.001
Longevity regulating pathway	0.001	0.008
AMPK signaling pathway	0.001	0.001
Neurotrophin signaling pathway	0.001	0.018
Ras signaling pathway	0.001	0.015
Human papillomavirus infection	0.001	0.022
Proteoglycans in cancer	0.002	0.002
Bacterial invasion of epithelial cells	0.002	0.001
Endocytosis	0.002	0.023
Regulation of actin cytoskeleton	0.002	0.003
Hippo signaling pathway	0.002	0.005
Phospholipase D signaling pathway	0.002	0.021
mTOR signaling pathway	0.002	0.008
Pathways in cancer	0.003	0.003
Cholinergic synapse	0.004	0.039
Thyroid hormone signaling pathway	0.006	0.002
Progesterone-mediated oocyte maturation	0.006	0.034
Autophagy	0.007	0.021
ErbB signaling pathway	0.007	0.034
Fc gamma R-mediated phagocytosis	0.007	0.002
Prolactin signaling pathway	0.007	0.005
Signaling pathways regulating pluripotency of stem cells	0.007	0.008
Colorectal cancer	0.009	0.001
Fluid shear stress and atherosclerosis	0.011	0.034
Non-small cell lung cancer	0.012	0.004
Endocrine resistance	0.012	0.028
Endometrial cancer	0.013	0.002
Pancreatic cancer	0.013	0.019
PI3 K-Akt signaling pathway	0.013	0.040
Human T-cell leukemia virus 1 infection	0.015	0.019
C-type lectin receptor signaling pathway	0.017	0.029
Gastric cancer	0.019	0.008
Insulin resistance	0.019	0.047
GABAergic synapse	0.023	0.039
Relaxin signaling pathway	0.023	0.039
Choline metabolism in cancer	0.023	0.006
Glioma	0.024	0.042
Adipocytokine signaling pathway	0.024	0.018
Type II diabetes mellitus	0.026	0.025
FoxO signaling pathway	0.031	0.039
T cell receptor signaling pathway	0.034	0.047
Cellular senescence	0.035	0.005
Phosphatidylinositol signaling system	0.035	0.016
B cell receptor signaling pathway	0.038	0.006
Hepatocellular carcinoma	0.039	0.003
Wnt signaling pathway	0.044	0.039

To further evaluate the shared methylation profiles of former and current cannabis smoking, we conducted an additional pathway analysis by selecting all DMPs that were identified in both the former and current cannabis smoking analyses. Overall, 94 percent of the overlapping DMPs were consistent in their effect direction (Beta FC). We identified 64 pathways enriched by the overlapping DMPs (Additional file 5) and compared these pathways to the individual analyses. Out of the 64, 46 pathways were also identified in both the individual analyses (former and current smoking), 4 were only identified in the individual analysis for former smoking, and 12 overlapped with the current smoking individual analysis. Only two pathways (circadian rhythm and tight junction) were unique to the overlapping DMG analysis.

Only two pathways, circadian rhythm and tight junction, were unique to the overlapping genes’ analysis. The remaining pathways were also identified in both former and current smoking analyses (46 pathways) or overlapped only with former (4 pathways) or current (12 pathways) cannabis smoking differentially methylated pathways.

## Discussion

In this study, we report three main observations. First, we determined that cannabis smoking is linked with numerous epigenome-wide changes. Second, we note that even with cannabis smoking cessation there remains significant blood epigenetic disruptions along thousands of genes. Third, these persistent methylation changes despite cannabis smoking cessation were highly enriched for aging- and cancer-related pathways. These observations indicate that the effect of smoking cannabis on the epigenome may be long lasting. Furthermore, our study shows the specific effects of cannabis smoking on epigenetic regulation in a cohort of older adults. This is of key importance due to the growing aging population, the increasing number of older adults using cannabis [29], and the lack of studies in this age group.

Our work adds to the literature on cannabis’s impact on the epigenome. Previous research has identified only statistically suggestive DMPs (*p* < 0.001) associated with cannabis use in a small cohort of young adults [13], while others have identified one DMP within the gene *CEMIP* in a cohort of women [30]*.* More recently, a couple of hundreds DMPs were reported to be associated with cannabis use in participants in the CARDIA cohort [31]; however, this investigation focused on young and middle aged adults, cannabis use by any form of consumption, and did not specifically evaluate the effects of smoking cessation. We note, however, that estimations of cannabis use (whether by joint-year, duration of use, recency of use) is quite different between our studies [13, 31], therefore between-study comparisons remain a challenge. Our analyses suggest that cannabis smoking has genome-wide consequences on blood DNA methylation of older adults and examined current as well as former cannabis smoking. Furthermore, we identified new genes and pathways associated with cannabis smoking and also replicated 85 DMGs (Additional file 6) and 3 differentially methylated pathways (dopaminergic synapse, human papillomavirus infection, and oxytocin signaling pathway) previously reported [31]. No specific CpGs were replicated.

Our analyses highlighted several genes with plausible links to the therapeutic effects of cannabis. *NEFM, GDAP,* and *JPH1* are among the most significant DMGs found in our analyses; these genes are located within CpG islands (regions of the genomes rich in CpGs that can highly influence downstream gene expression). Briefly, *GDAP* contributes to neuron function and maintenance [32]. *NEFM* is part of a dopamine receptor-interacting protein gene family that affects multiple aspects of dopamine receptor activity [33] and has been associated with response to antipsychotic medications [34] and in smoking initiation [35]. *JPH1* has an important signaling role in all excitable cell types, mainly in muscle and neural cells [36]. These three genes are furthermore implicated in a group of motor and sensory neuropathies called Charcot-Marie-Tooth Disease [32, 37, 38], which recently was shown to be effectively treated by cannabis to reduce pain and psychosocial stress [39]. The epigenetic regulation of *NEFM *[40]*, GDAP and JPH1* may contribute to the therapeutic effects of cannabis, specifically pain and stress relief. While epigenetic changes may partially explain some of the positive psychiatric and neurologic effects of cannabis, our study nonetheless also revealed epigenetic disruptions along genes that may influence cannabis’s more detrimental psychotropic effects. For example, we identified the type 1 cannabinoid receptor gene (*CNR1*) as a hypermethylated DMG in former cannabis smoking, the effect of DNA methylation on *CNR1* is not fully defined, however in the prefrontal cortex hypermethylation of *CNR1* is associated with lower gene expression [41]. *CNR1* is a key component of the cannabinoid system and the main target of tetrahydrocannabinol, the principal psychoactive ingredient of cannabis. *CNR1* expression is increased in patients with schizophrenia [42] and it has been suggested that certain alleles of this gene may increase the risk of cannabis use disorder [43]. Other research has shown a significant association between *CNR1* gene variations and decreased volume of the right anterior cingulum with cannabis exposure [44]. The *FAAH* gene was also identified in our study as being hypomethylated in current cannabis smoking compared to never smoking. *FAAH* encodes for the fatty acid amide hydrolase enzyme; animal models have shown that inhibition of this gene reduces the breakdown of endogenous cannabinoids and increases non-opioid-induced analgesia [45]. Specific polymorphisms in this gene are associated with cannabis dependence [42, 46]. Here, we propose that epigenetic alterations could also contribute to these associations.

Of concern in our analysis were the numerous enriched biological pathways that persisted despite cannabis smoking cessation. Aging-related pathways, for instance, continued to be epigenetically disrupted even in former cannabis smokers, echoing previous evidence that cannabinoids and in particular cannabidiol can induce cellular senescence. As an example, treatment of human Sertoli cells with cannabidiol inhibited cell proliferation and DNA synthesis, activated p53 signaling, and induced the expression of numerous senescence-associated secretory phenotype-related genes [47]. We also found cancer-related pathways to be highly enriched amongst the former and current cannabis smoking groups. Whether cannabis smoking increases the risk of developing cancer remains an ongoing subject of debate. Analyses of cannabis smoke have shown known carcinogens such as polycyclic aromatic hydrocarbons [48], while murine lung epithelial cells exposed to cannabis smoke demonstrate upregulation of genes associated with DNA damage response [49]. Nonetheless, a strong causal link between cannabis smoking and cancer has not been fully established in the clinical literature. A meta-analysis suggested low-strength evidence that cannabis smoking could be associated with the development of testicular germ cell tumors, but firm conclusions regarding its link with lung cancer and head and neck cancer could not be made [50]. Studies evaluating the link between cancer and cannabis smoking have likely been hampered by inconsistent reporting of cannabis habits and confounding by tobacco smoking. However, the findings from our study should raise concern that cannabis smoking may induce epigenetic injury of oncogenic potential.

Our study was limited by multiple factors. First, our sample size was small and did not allow us to directly compare the DNA methylation profiles of current and former cannabis smoking directly. Nevertheless, our analyses suggest that there may be a modest DNA methylation signature that differentiates former from current smoking. Second, without concurrent mRNA or protein readouts from the same individuals, we are unable to say whether the epigenetic disruptions associated with former or current cannabis smoking result in significant downstream alterations. Third, concurrent cannabis and tobacco use is often observed [51] and their independent effects on blood DNA methylation were not able to be assessed here due to sample size limitations. Future studies in larger cohorts stratified by both cannabis and cigarette smoking status would better distinguish their unique impacts on the blood methylome. However, we identified epigenetic disruptions associated with cannabis smoking that remained significant even after we adjusted for cigarette smoking status, suggesting that this cannabis-related epigenome signature is still somewhat independent of cigarette smoking. Fourth, our study would have been greatly enhanced by a longitudinal, repeated measures analysis that could have assessed the permanence of these findings with ongoing cannabis smoking or sustained cessation. Finally, cannabis smoking was self-reported in our study and collected during a time period when recreational cannabis smoking was still illegal in Canada. It is conceivable that the accuracy of self-reported smoking status may have been influenced by the legal standing of cannabis at the time.

## Conclusions

Despite these limitations, our findings importantly demonstrate that cannabis smoking can alter the circulating immune cell epigenome even after smoking cessation. The cannabis-related changes in DNA methylation may have downstream consequences in important aging- and cancer-related biological processes that could affect older adults who were part of our study population. With the growing popularity of cannabis, our research would suggest caution when it comes to cannabis smoking.

## Supplementary Information

Additional file 1

Additional file 2

Additional file 3

Additional file 4

Additional file 5

Additional file 6

## Data Availability

Data can be obtained from GEO DataSets, accession number GSE255929.

## Abbreviations

AMPK: 5'AMP-activated protein kinase
BMI: Body Mass Index
CanCOLD: Canadian Cohort Obstructive Lung Disease
CARDIA: Coronary Artery Risk Development in Young Adults
CEMIP: Cell Migration-Inducing and Hyaluronan-Binding Protein
CNR1: Cannabinoid Receptor 1
COPD: Chronic Obstructive Pulmonary Disease
CpGs: Cytosine-phosphate-Guanine
CYP4 F11: Cytochrome P450 Family 4 Subfamily F Member 11
DMP: Differentially Methylated Position
ErbB: Erythroblastic Leukemia Viral Oncogene Homolog
FAAH: Fatty Acid Amide Hydrolase
FDR: False Discovery Rate
FoxO: Forkhead Box O
GDAP: Ganglioside-Induced Differentiation-Associated Protein
IGLL1: Immunoglobulin Lambda Like Polypeptide 1
JMJD1 C: Jumonji Domain Containing 1 C
JPH1: Junctophilin 1
KEGG: Kyoto Encyclopedia of Genes and Genomes
KLF6: Krüppel-Like Factor 6
MAPK: Mitogen-Activated Protein Kinase
mTOR: Mammalian Target of Rapamycin
NEFM: Neurofilament Medium Polypeptide
PC: Principal Component
PCA: Principal Component Analysis
PI3 K-Akt: Phosphoinositide 3-Kinase – Protein Kinase B (Akt)
PLXDC2: Plexin Domain Containing 2
Rap1: Ras-Related Protein Rap- 1
WDR3: WD Repeat Domain 3
