# Supplementary material for: Cannabis smoking is associated with persistent epigenome-wide disruptions despite smoking cessation
Source: BMC Pulm Med. 2025 Apr 9;25:168. doi: 10.1186/s12890-025-03634-9 (PMC11980083; doi:10.1186/s12890-025-03634-9)
Supplement: Supplementary file 4 — Additional file 4 [file 12890_2025_3634_MOESM4_ESM.docx]

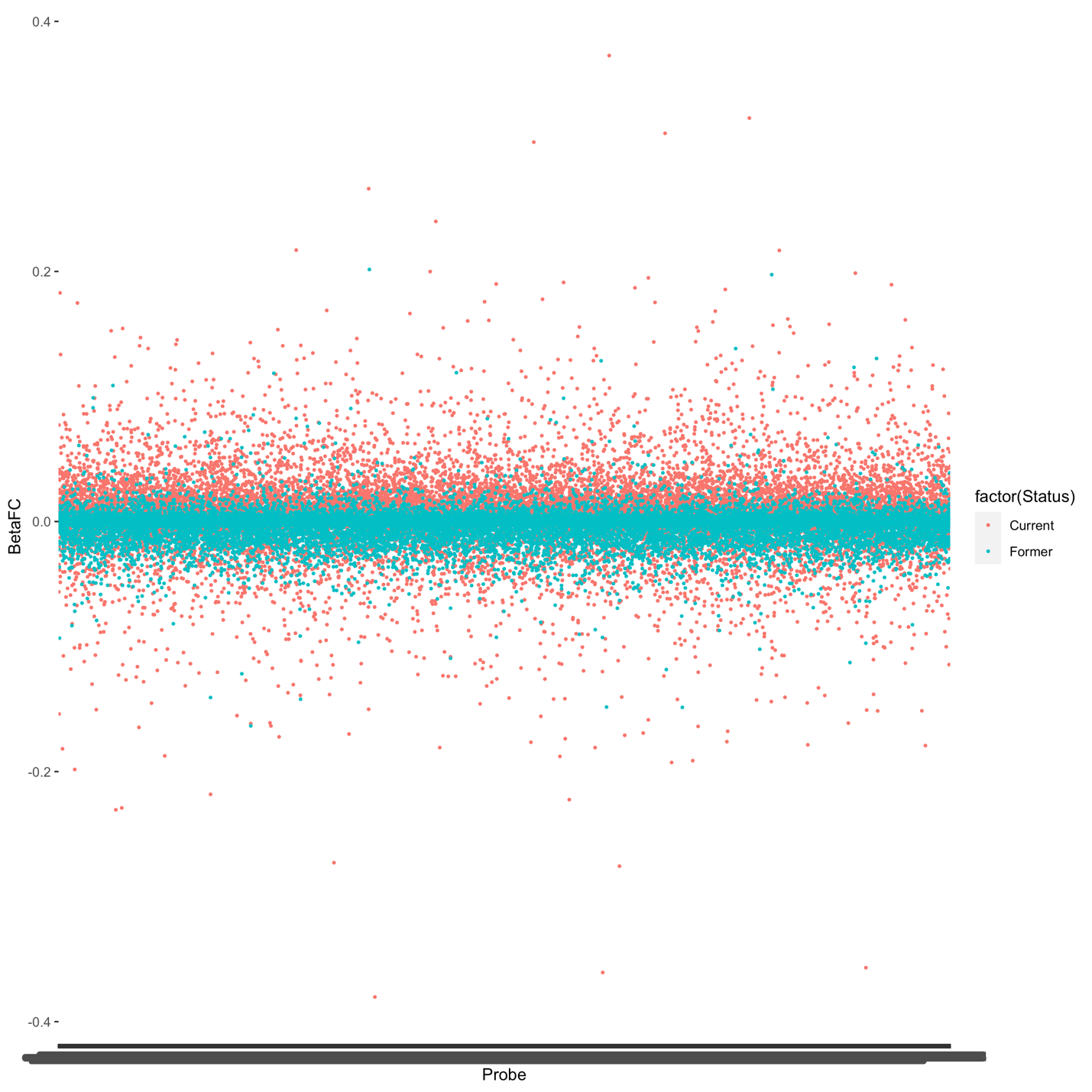


Additional figure 3. Effect size of differentially methylated positions (DMPs) associated with current (BetaFC=0.011 [0.004-0.025]) (pink dots) and former (Beta FC=0.003 [0.001-0.009]) (blue dots). X-axis represent each of the DMP identify. Y-axis correspond to DMPs effect sizes (Beta FC )


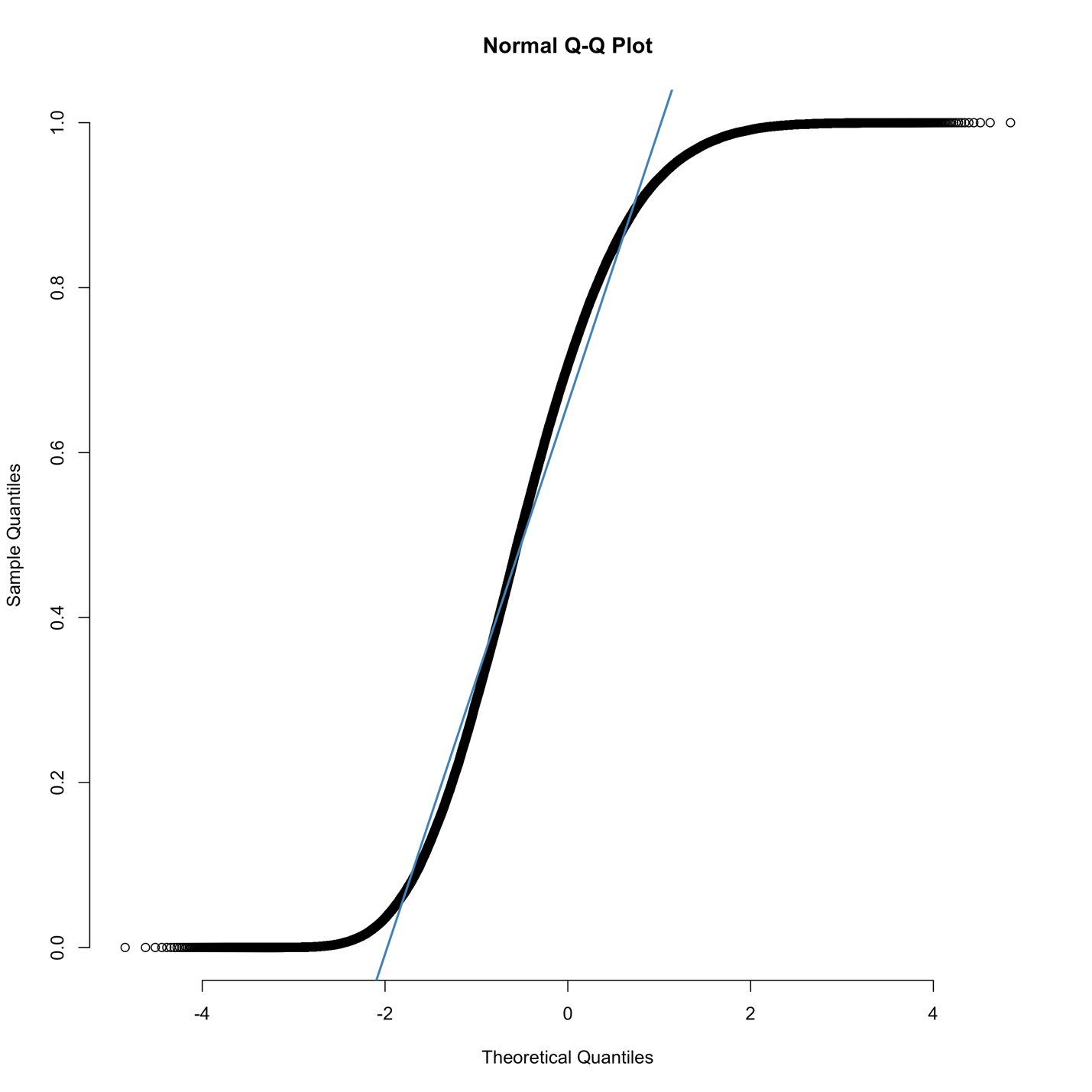


Additional figure 1. QQ-plot of the epigenome-wide analysis on former cannabis smoking. Lambda = 0.31


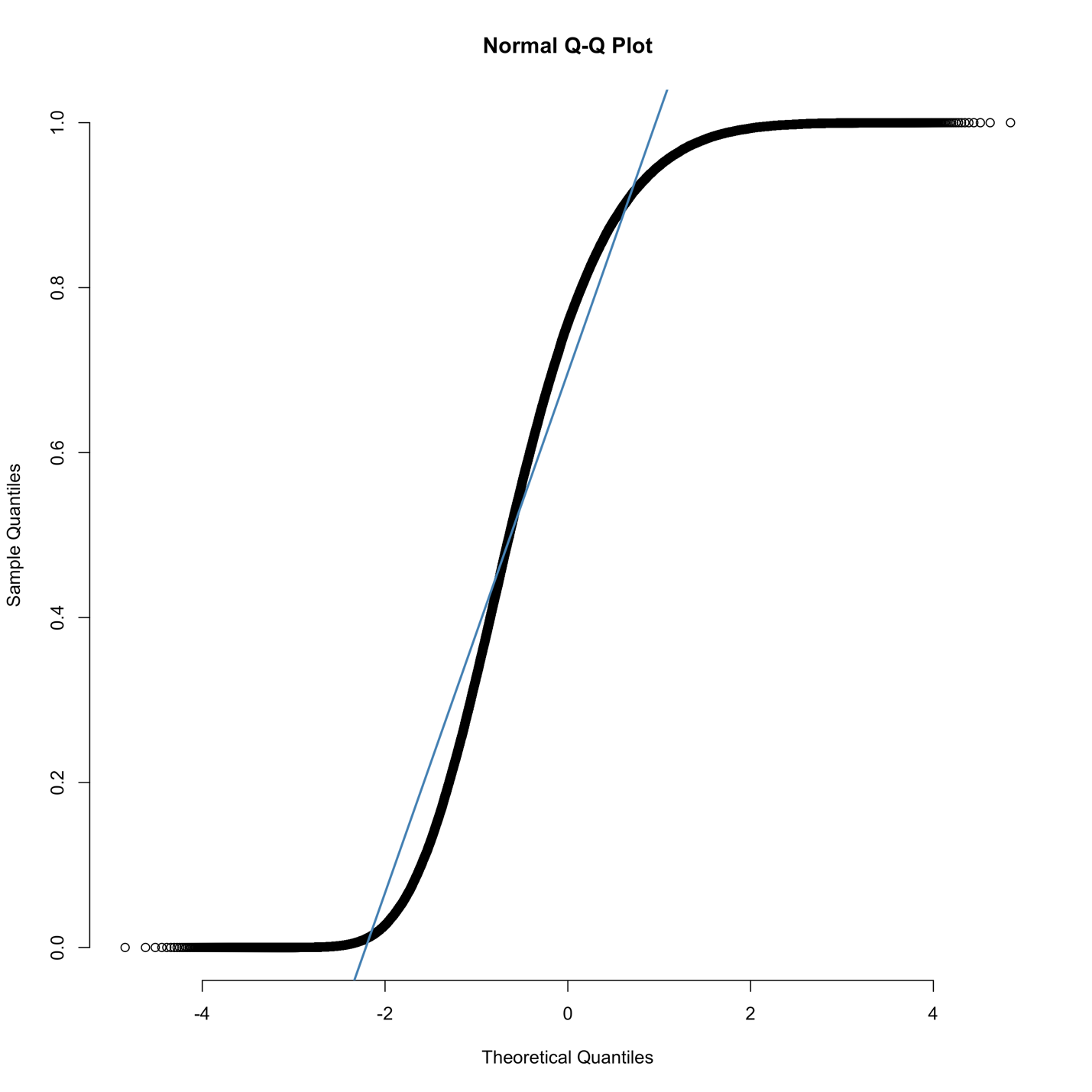


Additional figure 2. QQ-plot of the epigenome-wide analysis on current cannabis smoking. Lambda = 0.21
